# The *rolB* plant oncogene affects multiple signaling protein modules related to hormone signaling and plant defense

**DOI:** 10.1038/s41598-018-20694-6

**Published:** 2018-02-02

**Authors:** Victor P. Bulgakov, Yulia V. Vereshchagina, Dmitry V. Bulgakov, Galina N. Veremeichik, Yuri N. Shkryl

**Affiliations:** 1 Institute of Biology and Soil Science, Far Eastern Branch of the Russian Academy of Sciences, 159 Stoletija Str., Vladivostok, 690022 Russia; 2 Far Eastern Federal University, Vladivostok, 690950 Russia

## Abstract

The *rolB* plant oncogene of *Agrobacterium rhizogenes* perturbs many biochemical processes in transformed plant cells, thereby causing their neoplastic reprogramming. The oncogene renders the cells more tolerant to environmental stresses and herbicides and inhibits ROS elevation and programmed cell death. In the present work, we performed a proteomic analysis of *Arabidopsis thaliana rolB*-expressing callus line AtB-2, which represents a line with moderate expression of the oncogene. Our results show that under these conditions *rolB* greatly perturbs the expression of some chaperone-type proteins such as heat-shock proteins and cyclophilins. Heat-shock proteins of the DnaK subfamily were overexpressed in *rolB*-transformed calli, whereas the abundance of cyclophilins, members of the closely related single-domain cyclophilin family was decreased. Real-time PCR analysis of corresponding genes confirmed the reliability of proteomics data because gene expression correlated well with the expression of proteins. Bioinformatics analysis indicates that *rolB* can potentially affect several levels of signaling protein modules, including effector-triggered immunity (via the RPM1-RPS2 signaling module), the miRNA processing machinery, auxin and cytokinin signaling, the calcium signaling system and secondary metabolism.

## Introduction

Decades-long study of plant-*Agrobacterium* interactions and T-DNA oncogene function has revealed very complex behavior of these systems and a sophisticated mechanism of pathogenesis. The *rolB* oncogene of *Agrobacterium rhizogenes* is the gene associated with the largest number of disputes and conflicting opinions. Since 1991, it has gradually become apparent that *rolB* perturbs hormonal signaling pathways in transformed plants and plant cells^1,2^. RolB alters leaf and flower morphology, provokes heterostyly and the formation of adventitious roots in plant explants^3^, disturbs root geotropism^4^ and substantially increases the sensitivity of roots to auxin^1,5^. The *rolB* gene promotes *de novo* meristem formation in tobacco thin cell layers and plants^6,7^, and the type of organ that is formed from these meristems depends on many factors. The reproductive fate of the ovule and the process of anther dehiscence are also greatly affected in *rolB*-transformed plants^2,7,8^. Later, it was found that *rolB* influences ROS signaling and represses apoptosis in transformed calli^9,10^. These traits are associated with extremely high resistance of *rolB*-transformed callus cells to the ROS-inducing herbicides paraquat and menadione^9^. In general, *rolB* represses growth, and high expression of the gene causes cell necrosis^3^. However, cells transformed with moderately or weakly expressed *rolB* genes acquire a remarkable ability to resist various types of stress factors^11^ and display enhanced defense against pathogenic fungi^12^. The activation of secondary metabolism in *rolB*-transformed calli and plants is a known characteristic of *rolB*-transformed callus and plant cells, which reflects the general defensive status of the cells and appears to be most likely due to the activation of genes encoding the MYB and bHLH transcription factors^13–15^.

The first indication that a plant T-DNA oncoprotein might act as a chaperone was published in 2007^16^. The *6b* gene of *A. tumefaciens* belongs to the *plast* (RolB) gene family^17^. It was shown that the 6b oncoprotein binds to histone H3 and causes modification of transcription patterns in the host nuclei by changing the epigenetic status of the host chromatin^16–18^. By affecting chromatin structure, the histone chaperone activity of 6b regulates the expression of genes related to auxin and cytokinin biosynthesis^18^. In principle, histone modification is a widely known epigenetic alteration that occurs during animal and human oncogenesis^19^. Other chaperones, such as heat-shock proteins and cyclophilins, have been studied intensively for a long time to show their pivotal role in the processes of oncogenesis in human cells^20,21^.

Plants generate unorganized cell masses, such as callus or tumors, in response to stresses, such as wounding or pathogen infection^22^. This experimental system has been used extensively in basic research to address question how plants perceive and transduce endogenous and environmental signals and how they induce or maintain cell differentiation/dedifferentiation^22^. Presently, callus cultures are widely used in proteomic experiments as a universal model, giving a relatively standardized and homogeneous basis for research. Plant callus cultures were successfully used in proteomics experiments to study the molecular mechanisms underlying different aspects of cell differentiation and somatic embryogenesis^23–25^, stress adaptation^26^, and *Agrobacterium*-plant interaction^27^.

In this study, we used proteomics analysis in order to identify proteins with different expression in transformed calli. Transformed by the *rolB* gene calli are primary tumors, which can further differentiate in organs^2,6^. Taking into account that in *rolB*-transformed plants, the *rolB* signaling is interfered with the tissue-specific and developmental signaling^2,7,8^, we focused on studying primary tumors to see the first layer of regulation devoid of developmental signals. For this, we used moderately *rolB*-expressing *Arabidopsis thaliana* calli to find proteins whose abundance was significantly affected by the transformation. The term “moderately expressed” was initially introduced on the model of *Rubia cordifolia*^28^ and then applied to *Arabidopsis*^9^. The attribution to the average, weak or strong levels of *rolB*-gene expression was made based on a scale developed earlier^28^. According to this scale, the average expression of the gene is within 0.3–1.0 relative expression level units (that corresponds to expression of *rolB* in *rolABC*- or wild-type pRiA4-transformed cells). We use such callus line where the oncogene was expressed at physiological conditions. In this case, no signs of cell death or necrosis were observed. The culture grew well and the biomass accumulation was almost equal to the control vector-transformed culture. In other words, we created normal physiological conditions for tissues that express the oncogene.

Proteomics analysis showed that *rolB* perturbs the expression of chaperone-type proteins. In particular, heat-shock proteins of the DnaK subfamily were overexpressed in *rolB*-transformed calli, whereas the abundance of cyclophilins, members of the closely related single-domain cyclophilin family, decreased.

## Experimental Procedures

### Plant Material

*Arabidopsis thaliana* Columbia (Col-0) vector control and *rolB*-transgenic callus cultures were obtained from seedlings using the pPCV002-CaMVBT construct as described previously^9^. The calli were cultivated in W_2,4-D_ medium supplemented with 0.4 mg l^−1^ 2,4-dichlorophenoxyacetic acid in the dark at 24 °C with 30-day subculture intervals. Samples were taken from 21-day cultures designated as “At” (*A. thaliana* vector control) and “AtB-2” (*A. thaliana rolB*-transformed calli). These cultures were two years of age. Three biological experiments were carried out.

### 2-D Gel Electrophoresis and quantification of protein expression

Proteins were isolated from 0.5 g fresh weight of calli using a phenol extraction methanol/ammonium acetate precipitation method^29^. The phenolic phase was collected and precipitated overnight in five volumes of 100 mM ammonium acetate in ethanol at −20 °C. After centrifugation (10 min, 6000 g, 4 °C), the pellet was washed twice with ice-cold acetone.

For isoelectric focusing, dried protein pellets were dissolved in IPG buffer (9.5 M urea, 4% w/v CHAPS, 2% Pharmalyte pH 3–10 (GE Healthcare, Uppsala, Sweden), DeStreak Reagent (GE Healthcare) and 0.01% w/v bromophenol blue). Protein concentration was determined using an RC/DC kit (Bio-Rad Laboratories Inc., Hercules, CA, USA). A total of 500 µg of whole protein sample in 350 ml IPG buffer was applied to 18-cm Immobiline DryStrip pH 3–10 NL (GE Healthcare) by passive rehydration for 12 h at 20 °C according to the manufacturer’s recommendations. IEF was performed in a Protean IEF Cell (Bio-Rad) for 60,000 V-h. Before separation in the second dimension, the Immobiline DryStrip was equilibrated in buffer (6 M urea, 0.375 M Tris-HCl, pH 8.8, 2% SDS, 20% glycerol, and 2% DTT) for 10 min. For SDS-PAGE, 12% polyacrylamide gels with 4% stacking gels were run in a Protean II xi cell (Bio-Rad). The gels were stained with Coomassie Brilliant Blue G-250. Three control and three experimental gels were used in the analysis.

#### Protein Expression

Gels were scanned using the PharosFX Plus System (Bio-Rad). PDQuest 8.0.1 Advanced software (Bio-Rad) was used for image and analysis of protein maps. The Spot Detection Wizard was used to select the parameters for spot detection, such as a faint spot and a large spot cluster. The results of automated spot detection were checked and manually corrected. On average, 1,500 protein spots were detected on gels of *Arabidopsis* calli. A local regression model (Loess) was used for normalization of spot intensity. The protein expression was accessed using PDQuest 8.0.1 Advanced software and was presented as mean total intensity of a defined spot in a replicate gel group. Spot quantity is the sum of the intensities of pixels inside the boundary. Fold of protein expression change was accessed based on mean protein intensity. For quantitative differentiation, a 1.5-fold change or higher in the average spot intensity was regarded as significant. Statistical significance of differences was assessed using Student’s *t* test at a significance level of 0.05 in three replicates. All identified proteins in qualitatively different spots were considered. Mean expression values and standard deviations were calculated from three biological experiments.

### Mass spectrometry

The total number of samples analyzed by MALDI was 203. The number of technical replicates was 2–3 (up to 8 for important proteins). Individual protein spots selected on the basis of image-analysis output were excised and digested in-gel with trypsin (Trypsin V511, Promega, Madison, WI, USA) as previously described^30^. For MALDI-TOF identification, 0.5–1 μl of the sample (50% solution of acetonitrile in water, 0.1% TFA) was placed on a ground steel MALDI target plate or AnchorChip or SmallAnchor (depending on the protein quantity; also see Supplementary Dataset S1), and 0.5–1 μl of the matrix (α-cyano-4-hydroxycinnamic acid) was added. For LC-ESI-MS/MS, 10-μl protein samples dissolved in water containing 0.1% TFA were used.

#### MALDI-TOF Mass Spectrometry and Protein Identification

All mass spectra were acquired with an Autoflex (Bruker Daltonics, Bremen, Germany) MALDI-TOF mass spectrometer with a nitrogen laser operated in the positive reflector mode (standard method RP 700–3500 Da.par) under the control of FlexControl software (version 3.4; Bruker Daltonics). The analysis was performed in the automatic mode (AutoXecute – automatic Run). The spectra were externally calibrated using the CalibratePeptideStandards.FAMSMethod and a standard calibration mixture (Protein Calibration Standard I, Bruker Daltonics). The data files were transferred to Flexanalysis software version 3.4 (Bruker Daltonics) for automated peak extraction. Assignment of the first monoisotopic signals in the spectra was performed automatically using the signal detection algorithm SNAP (Bruker Daltonics). For MS and MS/MS analyses, we used the PMF.FAMSMethod and SNAP_full_process.FALIFTMethod, respectively. Each spectrum was obtained by averaging 1500–5000 laser shots (300 shots in a step) acquired at the minimum laser power. The data were analyzed using BioTools (version 3.2; Bruker Daltonics). A peptide mass tolerance of 0.5 Da and a fragment mass tolerance of 0.5 Da were adopted for database searches. The *m*/*z* spectra were searched against the *Arabidopsis thaliana* NCBInr and SwissProt databases using the Mascot search engine. Threshold score was 40. Further data were analyzed using UniProt (http://www.uniprot.org/uniprot/) and other specialized databases and programs as indicated below. The mass spectrometry proteomics data have been deposited to the ProteomeXchange Consortium via the PRIDE^31^ partner repository with the dataset identifier PXD005889 (DOI: 10.6019/PXD005889).

#### LC-ESI-MS/MS

For determination of proteins of low abundance, we used (in additional to MALDI analysis) an HCTultra PTM Discovery System (Bruker Daltonik GmbH, Germany) equipped with a Proxeon EASY-nLC ultra-performance liquid chromatograph and a nanoFlow ESI sprayer. The coupling of Proxeon EASY-nLC to the Bruker HCT ion trap was performed using the program HyStar v3.2 (Bruker Daltonik GmbH). The HCTultra is equipped with a high-capacity ion trap that enables the acquisition of MS/MS data on low-abundance precursor ions. For the LC studies, Buffer A (0.1% formic acid in water) and Buffer B (0.1% formic acid in acetonitrile/10% water) (Acetonitrile G Chromasolv for HPLC, super gradient grade; Sigma-Aldrich, Steinheim, Germany) were used. Separation was carried out on a C18-reversed phase EASY-Column (10 cm × 75 µm i.d., 3-µm beads, 120-Å pore size, Thermo Fisher Scientific). The flow rate was 300 nl min^−1^ with the following gradient: 5% Buffer B at 0 min, linearly increased to 35% B at 10 min and to 100% B from 10 to 25 min followed by washing at 100% B from 25 to 40 min. The ion trap capillary temperature was set to 300 °C, and the dry gas flow was 5 l min^−1^. The ion trap was set to acquire in positive ion mode, scanning in the manufacturer-specified standard enhanced mode (8,100 *m/z*/s) between *m/z* 300 and 2,000 for MS, averaging five spectra, and accumulated 200,000 charges (by ion charge control). Collision-induced dissociation fragmentation was performed on the four most intense ions with the threshold for precursor ion selection at an absolute intensity of 20,000. The strict active exclusion was used; a precursor ion was excluded after one spectrum and released after 0.1 min. MS-MS spectra were scanned from *m/z* 300–2,000, averaging three spectra. Data were analyzed using BioTools (version 3.2; Bruker Daltonics). The following parameters were used for database searches: peptide mass tolerance 0.1% and fragment mass tolerance 0.5 Da.

### RNA isolation, cDNA synthesis and real-time PCR

The isolation of total RNA and first-strand cDNA synthesis were carried out as described previously^9^. RNA samples were isolated from callus cultures during the linear phase of growth (20–22 days). RNA concentration and 28S/18S ratios were determined using an RNA StdSens LabChip® kit and Experion^TM^ Automated Electrophoresis Station (Bio-Rad Laboratories Inc., Hercules, CA, USA) with Experion^TM^ Software System Operation and Data Analysis Tools (version 3.0) following the manufacturer’s protocol and recommendations. The samples with 28S/18S ribosomal RNA between 1.5–2.0 and an RNA Quality Indicator (RQI) above 9.0 were used for real-time PCR analysis. Quantitative real-time PCR (qPCR) analysis was performed using a CFX96 (Bio-Rad Laboratories, Inc., Hercules, CA, USA) with 2.5 × SYBR green PCR master mix containing ROX as a passive reference dye (Syntol, Russia) as described^9^. Two biological replicates, resulting from two different RNA extractions, were used for analysis, and three technical replicates were analysed for each biological replicate. The gene-specific primer pairs used in the qPCR were as follows in the Supplementary Table S1. *A*. *thaliana* actin (*AtAct2*) and ubiquitin (*AtUBQ10*) genes (GenBank ac. no. NM_112764 and NM_001084884, respectively) were used as endogenous controls to normalize variance in the quality and the amount of cDNA used in each real-time RT-PCR experiment^9^. The highest expressing sample assigned the value 1 in the relative mRNA calculation using the formula 2^−ΔΔCT^. Data were analyzed using CFX Manager Software (Version 1.5; Bio-Rad Laboratories, Inc.). For comparison among multiple data, analysis of variance (ANOVA) followed by a multiple comparison procedure was employed. Fisher’s protected least significant difference (PLSD) *post-hoc* test was employed for the inter-group comparison. A difference of *P* < 0.05 was considered significant.

### Protein Network Visualization

The network was built using the program Cytoscape as previously described^32^. The data loaded into the program were obtained from the PAIR [PAIR-V3.3^33^, http://www.cls.zju.edu.cn/pair/]. The protein-protein interactions presented in PAIR were compared with the databases BioGRID^34^ (http://thebiogrid.org/). The size of each circle is correlated with the “betweenness centrality” metric, which describes the global position (“centrality”) of the protein in the interactome. Betweenness centrality was calculated by Cytoscape. Information about protein-protein interactions was also obtained by using UniProt and by linking Cytoscape with external databases (IntAct and STRING). The network was validated using recently developed algorithms^35,36^.

### Data Availability

The mass spectrometry data have been deposited to ProteomeXchange via the PRIDE partner repository with the dataset identifier PXD005889 (Project DOI: 10.6019/PXD005889).

## Results

### Characterization of the *rolB*-expressing line AtB-2

Expression of *rolB* in AtB-2 callus line was tested by qPCR before proteomic analysis. The *rolB*-expressing callus line AtB-2 was shown to be a line with moderate *rolB* expression (0.56 ± 0.04 relative expression level units, see Supplementary Table 2). The presence of the RolB protein in callus extracts was confirmed by mass spectrometry (Supplementary Dataset S3).

### Proteomics Analysis

Total protein fractions were isolated from *Arabidopsis thaliana* vector control and *rolB*-transgenic callus cultures as described in Materials and Methods. Overall, 1,500 proteins were resolved on 2-D gels (Supplementary Figure S1). Of these, over 200 were identified using MALDI MS. Proteins whose determinations represented reliable data meeting the requirements of precision mass-spectrometric analysis and quantitative differences for proteins are included in Tables 1 and 2 and were considered in further analysis (see also Supplementary Dataset S1 and Dataset S2). Three differentially expressed proteins remain undetermined because the search of databases yielded no results. Thus, 31 proteins were upregulated in *rolB*-expressing cells compared with control cells (Table 1), and 29 proteins were down-regulated (Table 2). We also performed a targeted search of chaperone-type proteins using their predicted masses and isoelectric points. These data are presented in Table 3. To identify low-abundance proteins such as VH1-interacting kinase and heat stress tolerant DWD1 (DWD1/HTD1), we used an Anchor chip or SmallAnchor chip (otherwise, it was not possible to identify these proteins). Examples are shown in Supplementary Dataset S1. RolB itself was also detected (Supplementary Dataset S3).

**Table 1:** Proteins upregulated in *rolB*-expressing Arabidopsis calli.

	UniProtKB code	Name of the protein	Function or biological process	Activation, folds*	Notes^1^
1	Q9SN86 (MDHP_ARATH)	Malate dehydrogenase, chloroplastic/MDH	Carbohydrate metabolic process Tricarboxylic acid cycle	14 ± 2	Primary metabolism Response to cold
2	Q9LDV4 (ALAT2_ARATH)	Alanine aminotransferase 2, mitochondrial	Synthesizes pyruvate from L-alanine Photosynthesis	12 ± 2	Primary metabolism Response to hypoxia
3	Q9LVW7 (CARA_ARATH)	Carbamoyl-phosphate synthase small chain, chloroplastic	Amino-acid biosynthesis	10 ± 0.5	Primary metabolism Response to phosphate starvation
4	Q9LZ66 (SIR_ARATH)	Assimilatory sulfite reductase (ferredoxin), chloroplastic	Assimilatory sulfate reduction pathway during both primary and secondary metabolism	10 ± 2	Secondary metabolism Response to cold
5	Q9S7B5 (THRC1_ARATH)	Threonine synthase 1, chloroplastic	L-threonine biosynthesis	1 ± 0.5	Primary metabolism Stress-inducible^1^
6	P24102 (PER22_ARATH)	Peroxidase 22	Hydrogen peroxide catabolic process	5.6 ± 0.7	Plant defense
7	Q9SJZ2 (PER17_ARATH)	Peroxidase 17	Hydrogen peroxide catabolic process	3.4 ± 0.6	Plant defense
8	Q9C6Z3 (ODPB2_ARATH)	Pyruvate dehydrogenase E1 component subunit beta-2, chloroplastic	Fatty acid biosynthetic process Glycolysis	5.3 ± 0.7	Primary metabolism
9	Q9LFG2 (DAPF_ARATH)	Diaminopimelate epimerase, chloroplastic	Amino-acid biosynthesis	4 ± 1.5	Primary metabolism Stress-inducible^1^
10	Q9SRY5 (GSTF7_ARATH)	Glutathione S-transferase F7	Defense response to bacterium Defense response to fungus, incompatible interaction Response to salt stress	3.5 ± 0.4	Plant defense
11	P42760 (GSTF6_ARATH)	Glutathione S-transferase F6	Defense response to bacterium Response to oxidative stress Response to salt stress Response to water deprivation	3.5 ± 0.6	Plant defense Involved in camalexin biosynthesis
12	Q9FUS6 (GSTUD_ARATH)	Glutathione S-transferase U13	Detoxification Stress response	3 ± 0.2	Plant defense
13	Q9FWR4 (DHAR1_ARATH)	Glutathione S-transferase DHAR1, mitochondrial	Scavenging of ROS under oxidative stresses	2 ± 0.3	Plant defense Key component of the ascorbate recycling system
14	Q9STW6 (HSP7F_ARATH)	Heat shock 70 kDa protein 6, chloroplastic/Hsp70-6 Synonym: cpHsc70-1	Host-virus interaction, protein transport, stress response	2.4 ± 0.15	Chaperone
15	Q9LTX9 (HSP7G_ARATH)	Heat shock 70 kDa protein 7, chloroplastic/Hsp70-7	Host-virus interaction, protein transport, stress response	2.9 ± 0.6	Chaperone
16	Q9SIF2 (HS905_ARATH)	Heat shock protein 90-5, chloroplastic Hsp90-5/CR88 Synonym**:** Hsp88.1	Response to heat Response to salt stress Response to water deprivation Embryo development	2.4 ± 0.7	Chaperone
17	P21238 (CPNA1_ARATH)	Chaperonin 60 subunit alpha 1, chloroplastic, Cpn60	Chloroplast organization Embryo development	2.3 ± 0.6	Chaperone
18	O65282 (CH20_ARATH)	20 kDa chaperonin, chloroplastic, Cpn10	Stress response Required to activate the iron superoxide dismutases (FeSOD)	2 ± 0.6	Chaperone Functions along with Cpn60
19	O04130 (SERA2_ARATH)	D-3-phosphoglycerate dehydrogenase 2, chloroplastic	Amino-acid biosynthesis	2.5 ± 1	Primary metabolism
20	Q9M9K1 (PMG2_ARATH)	2,3-bisphosphoglycerate-independent phosphoglycerate mutase 2	Glycolysis	2.1 ± 0.5	Primary metabolism
21	Q42592 (APXS_ARATH)	L-ascorbate peroxidase S, chloroplastic/mitochondrial	Plays a key role in hydrogen peroxide removal	2 ± 0.5	Plant defense
22	Q9XI87 (Q9XI87_ARATH)	VH1-interacting kinase (VIK)	Auxin-activated signaling pathway, negative regulation of programmed cell death, plant-type hypersensitive response, response to cold and water deprivation	2 ± 0.6	Signal transduction, MAPK cascade
23	Q9ZUN8 (Q9ZUN8_ARATH)	HEAT STRESS TOLERANT, DWD1 Synonym: HTD1/WD-40 repeat family protein	Cul4-RING E3 ubiquitin ligase complexHeat stress response	2 ± 0.4	Signal transduction
24	Q9S7D8 (APS4_ARATH)	ATP sulfurylase 4, chloroplastic/APS4	Hydrogen sulfide biosynthetic process Regulation of hypersensitive response	2.2 ± 1.0	Positive regulation of flavonoid biosynthesis
25	Q9SA34 (IMDH2_ARATH)	Inosine-5′-monophosphate dehydrogenase 2	Purine biosynthesis	2.1 ± 1.0	Primary metabolism
26	P93819 (MDHC1_ARATH)	Malate dehydrogenase, cytoplasmic 1	Tricarboxylic acid cycle	2 ± 0.6	Primary metabolism Stress-inducible^1^
27	P57106 (MDHC2_ARATH)	Malate dehydrogenase, cytoplasmic 2	Tricarboxylic acid cycle	2 ± 0.7	Primary metabolism Stress-inducible^1^
28	Q94JQ3 (GLYP3_ARATH)	Serine hydroxymethyltransferase 3, chloroplastic	Glycine metabolic process	2 ± 0.2	Primary metabolism
29	O22832 (STAD7_ARATH)	Acyl-[acyl-carrier-protein] desaturase 7, chloroplastic, FAB2	Fatty acid biosynthetic process	2 ± 0.3	Plant defense
30	P41088 (CFI1_ARATH)	Chalcone-flavonone isomerase 1/TRANSPARENT TESTA 5	Flavonoid biosynthesis	2 ± 0.15	Secondary metabolism
31	Q8VY84 (KCY1_ARATH)	Probable UMP-CMP kinase 1	Pyrimidine nucleotide biosynthetic process	1.8 ± 0.12	Primary metabolism

^1^Data from UniProt and TAIR.

^2^Less and Galili^59^.

*Mean ± standard deviation of three biological repeats.

**Table 2:** Proteins down-regulated in *rolB*-expressing Arabidopsis calli.

	UniProtKB code	Name of the protein	Function or biological process^1^	Inhibition, folds	Notes^1^
1	Q9M885 (RS72_ARATH)	40S ribosomal protein S7-2	Structural constituent of ribosome	15 ± 4	Protein biosynthesis
2	Q9C514 (RS71_ARATH)	40S ribosomal protein S7-1	Structural constituent of ribosome	12 ± 2	Protein biosynthesis
3	P57720 (AROC_ARATH)	Chorismate synthase, chloroplastic	Catalyzes the last common step of the biosynthesis of aromatic amino acids, produced via the shikimic acid pathway	11 ± 2	Aromatic amino acid biosynthesis
4	Q38867 (CP19C_ARATH)	Peptidyl-prolyl cis-trans isomerase CYP19-3/Rotamase cyclophilin-2, ROC2	Protein folding	10 ± 0.5	Chaperone Signal transduction
5	P34791 (CP20C_ARATH)	Peptidyl-prolyl cis-trans isomerase CYP20-3/Rotamase cyclophilin-4, ROC4	Protein peptidyl-prolyl isomerization Links light and redox signals	10 ± 1	Chaperone
6	P34790 (CP18C_ARATH)	Peptidyl-prolyl cis-trans isomerase CYP18-3, ROC1	Protein peptidyl-prolyl isomerization Plant defense Hypersensitive response	6.6 ± 0.7	Chaperone
7	Q9SKQ0 (CP19B_ARATH)	Peptidyl-prolyl cis-trans isomerase CYP19-2/ROC6	Protein peptidyl-prolyl isomerization	6.0 ± 0.4	Chaperone
8	Q9ASS6 (PNSL5_ARATH)	Peptidyl-prolyl cis-trans isomerase CYP20-2	Protein peptidyl-prolyl isomerization NAD(P)H dehydrogenase complex assembly	3.4 ± 0.4	Chaperone Modulates the conformation of BZR1
9	Q96255 (SERB1_ARATH)	Phosphoserine aminotransferase 1, chloroplastic	Amino-acid biosynthesis	8.9 ± 1.0	Primary metabolism
10	Q93ZC5 (AOC4_ARATH)	Allene oxide cyclase 4, chloroplastic	Jasmonic acid biosynthetic process	5.4 ± 0.5	
11	O49485 (SERA1_ARATH)	D-3-phosphoglycerate dehydrogenase 1, chloroplastic	L-serine biosynthetic process Embryo development Pollen development	2.6 ± 0.5	Primary metabolism
12	Q8RWV0 (TKTC1_ARATH)	Transketolase-1, chloroplastic	Pentose-phosphate cycle	2.8 ± 0.3	Primary metabolism
13	Q9C5Y9 (Q9C5Y9_ARATH)	Initiation factor 3 g	Stimulates binding of mRNA and methionyl-tRNAi to the 40S ribosome	2.6 ± 0.2	Protein biosynthesis
14	Q8LFK2 (Q8LFK2_ARATH)	Adenine nucleotide alpha hydrolases-like protein	Hydrolase activity	2.7 ± 0.2	Response to stress
15	Q9FMF5 (RPT3_ARATH)	Root phototropism protein 3, RPT3	Substrate-specific adapter of an E3 ubiquitin-protein ligase complex (CUL3-RBX1-BTB)	2.2 ± 0.5	Signal transduction
16	O49203 (NDK3_ARATH)	Nucleoside diphosphate kinase III, chloroplastic/mitochondrial	Nucleoside triphosphate biosynthetic process	2.1 ± 0.5	Nucleotide metabolism
17	O04310 (JAL34_ARATH)	Jacalin-related lectin 34	Copper ion binding Response to cold	2.1 ± 0.5	Brassinosteroid biosynthetic process
18	Q8LBZ7 (SDHB1_ARATH)	Succinate dehydrogenase [ubiquinone] iron-sulfur subunit 1, mitochondrial	Tricarboxylic acid cycle	2.1 ± 0.2	Primary metabolism
19	Q8W4S6 (INV6_ARATH)	Beta-fructofuranosidase, insoluble isoenzyme CWINV6	Carbohydrate metabolic process	2 ± 0.15	Primary metabolism
20	F4JGR5 (PFPB2_ARATH)	Pyrophosphate-fructose 6-phosphate 1-phosphotransferase subunit beta 2	Glycolysis	2 ± 0.6	Primary metabolism
21	Q9LZT4 (EXLA1_ARATH)	Expansin-like A1	Plant-type cell wall loosening, unidimensional cell growth	2 ± 1	
22	O80713 (SDR3A_ARATH)	Short-chain dehydrogenase reductase 3a	Hypersensitive response	2 ± 0.2	Plant defense
23	Q9ZV34 (Q9ZV34_ARATH)	Pathogenesis-related thaumatin-like protein	Unknown	2 ± 1	Probably a defensive function
24	Q9LK23 (G6PD5_ARATH)	Glucose-6-phosphate 1-dehydrogenase, cytoplasmic isoform 1	Pentose-phosphate cycle	1.8 ± 0.4	Primary metabolism
25	P32962 (NRL2_ARATH)	Nitrilase 2	Indoleacetic acid biosynthetic process	1.8 ± 0.3	
26	Q9XEE2 (ANXD2_ARATH)	Annexin D2	Calcium-dependent phospholipid binding Response to stress	1.5 ± 0.2	Polysaccharide transport
27	Q9SR13 (FLK_ARATH)	Flowering locus K homology domain	RNA binding	1.5 ± 0.3	Positive regulation of flower development
28	O24456 (GBLPA_ARATH)	Receptor for activated C kinase 1 A, RACK1A	MAP-kinase scaffold activity Protein complex scaffold Signal transducer activity	1.5 ± 0.2	Signal transduction
29	Q9FWA3 (6GPD3_ARATH)	6-phosphogluconate dehydrogenase, decarboxylating 3	Pentose phosphate pathway	1.5 ± 0.2	Primary metabolism

^1^Data from UniProt and TAIR.

**Table 3:** Chaperone-type proteins which abundance was not changed in *rolB*-expressing calli.

	UniProtKB code	Name of the protein	Function or biological process	Notes
1	Q9LDZ0 (HSP7J_ARATH)	Heat shock 70 kDa protein 10, mitochondrial (Hsp70-10)	Response to heat Response to salt stress Response to virus	Chaperone
2	Q9S7C0 (HSP7O_ARATH)	Heat shock 70 kDa protein 14, cytoplasmic and nucleolar (Hsp70-14)	Response to heat	Chaperone
3	F4HQD4 (HSP7P_ARATH)	Heat shock 70 kDa protein 15 cytoplasmic and nucleolar (Hsp70-15)	Response to stress	Chaperone
4	P55737 (HS902_ARATH)	Heat shock protein 90-2, cytoplasmic (Hsp90-2) Synonym: Hsp81-2	Response to stress Defense response to bacterium	Chaperone Maintains appropriate levels of immune receptor proteins to avoid autoimmunity
5	Q9LV21 (TCPD_ARATH)	TCP-1/cpn60 chaperonin family protein, cytoplasmic (T-complex protein 1 subunit delta)	Folding of actin and tubulin	Chaperone

### Proteins Upregulated in *rolB*-expressing Cells

#### Primary metabolism and ROS-detoxifying enzymes

Several proteins involved in various biosynthetic processes of primary metabolism were strongly activated; these included alanine aminotransferase, carbamoyl phosphate synthase, malate dehydrogenase, threonine synthase, pyruvate dehydrogenase and others (Table 1). Another subset of upregulated proteins was represented by defensive enzymes involved in ROS metabolism. Among them were peroxidases, the activation of which in *rolB*-expressing cells was previously demonstrated at the level of gene expression^37^. The increase in expression of antioxidant enzymes determined in the present work was essentially the same as determined previously by other methods^38^, thus confirming the reliability of the proteomics experiments. The previously found induction of ascorbate peroxidase genes in *rolB*-transformed cells^9^ was also confirmed (Table 1). New data were obtained regarding glutathione S-transferases. Glutathione S-transferases F6 and F7, as well as glutathione S-transferase DHAR1, a key component of the ascorbate recycling system^39^, were upregulated in *rolB*-transformed cells. These transferases are involved in redox homeostasis and especially in the scavenging of ROS under oxidative stress conditions subsequent to induction by biotic or abiotic inducers^39^. Taken together, our data confirm the hypothesis^9^ that *rolB* affects ROS metabolism by participating in a cellular process that resembles the process of stress acclimation.

#### Heat-shock proteins and chaperonins

Heat-shock 70-kDa proteins 6 and 7 (Hsp70-6 and Hsp70-7), Hsp90-5, 20-kDa chaperonin (Cpn10) and chaperonin 60 subunit α1 were activated in *rolB*-expressing Arabidopsis cells (Table 1). It is known that in cooperation with other chaperones, Hsp70s stabilize preexisting proteins against aggregation and mediate the folding of newly translated polypeptides in the cytosol as well as within organelles^40^. Transgenic *Arabidopsis* plants expressing a fungal *hsp70* gene exhibited enhanced tolerance to heat stress and to osmotic, salt and oxidative stresses^40^.

The Hsp70 protein family is divided into two subfamilies: DnaK and Hsp110/SSE^41^. Of the DnaK subfamily, only chloroplastic AtHsp70-6 and AtHsp70-7 were upregulated in *rolB*-transformed cells. Other Hsp70 proteins found by the targeted analysis, such as AtHsp70-10 (DnaK subfamily, mitochondrial) and AtHsp70-14 and AtHsp70-15 (Hsp110/SSE subfamily, cytosolic), were found in equal abundance in control and *rolB*-expressing calli (Table 3).

#### Other proteins

The abundance of the VH1-interacting kinase (VIK, VH1-interacting tetratricopeptide repeat (TPR)-containing protein) in *rolB*-transformed cells was also increased. Another protein upregulated in the transformed cells was DWD1/HTD1. This protein was shown to participate in heat stress responses, possibly by interacting with Hsp90-1^42^. Enzymes that participate in secondary metabolism, such as chalcone-flavonone isomerase 1 and ATP sulfurylase 4, were upregulated.

### Proteins Down-Regulated in *rolB*-expressing Cells

Expression of the 40S ribosomal proteins S7-2 and S7-1 was significantly inhibited in *rolB*-expressing cells (Table 2). These proteins are structural constituents of the ribosome and participate in ribosomal RNA processing, ribosomal small subunit biogenesis and translation (BioGrid). Initiation factor 3 g was also down-regulated. This factor is involved in protein synthesis; together with other initiation factors, it stimulates binding of mRNA and methionyl-tRNAi to the 40S ribosome. These data indicate that *rolB* can potentially inhibit protein biosynthesis.

The expression of several enzymes involved in processes of primary metabolism such as glycolysis, the pentose phosphate cycle, amino acid biosynthesis, carbohydrate metabolic processes, the Calvin cycle and the Krebs cycle was moderately inhibited. Among them were chorismate synthase, phosphoserine aminotransferase 1, D-3-phosphoglycerate dehydrogenase 1, transketolase 1, glucose-6-phosphate 1-dehydrogenase, 6-phosphogluconate dehydrogenase, pyrophosphate-fructose 6-phosphate 1-phosphotransferase and others (Table 2). The suppression of these enzymes reflects the repressive action of *rolB* on primary metabolism that eventually causes the well-known growth-inhibiting effect of *rolB*.

Several peptidyl-prolyl cis-trans isomerases were down-regulated in *rolB*-transformed cells. This group of proteins, also called cyclophilins or immunophilins, has been shown to possess peptidyl-prolyl cis-trans isomerase (PPIase) activity that is involved in protein folding. It includes CYP18-3 (ROC1), CYP19-2 (ROC6), CYP19-3 (ROC2), CYP20-2 and CYP20-3 (ROC4). *RolB* affects only one family of closely related single-domain cyclophilins (Clade I)^43^ (Table 2). The receptor for activated C kinase 1 A (RACK1A) was down-regulated in *rolB*-transformed cells (Table 2). RACK1A, a WD-40-type scaffold protein, is the major RACK1 regulatory protein conserved in eukaryotes. RACK1A participates in multiple signal transduction pathways, including pathways mediated by RACK1A-cyclophilin interactions^44,45^. Interestingly, some enzymes involved in plant hormone biosynthesis, such as allene oxide cyclase 4 (jasmonic acid biosynthesis), jacalin-related lectin 34 (brassinosteroid biosynthesis) and nitrilase 2 (indoleacetic acid biosynthesis), were down-regulated (Table 2).

### Analysis of Gene Expression

**Figure 1:**
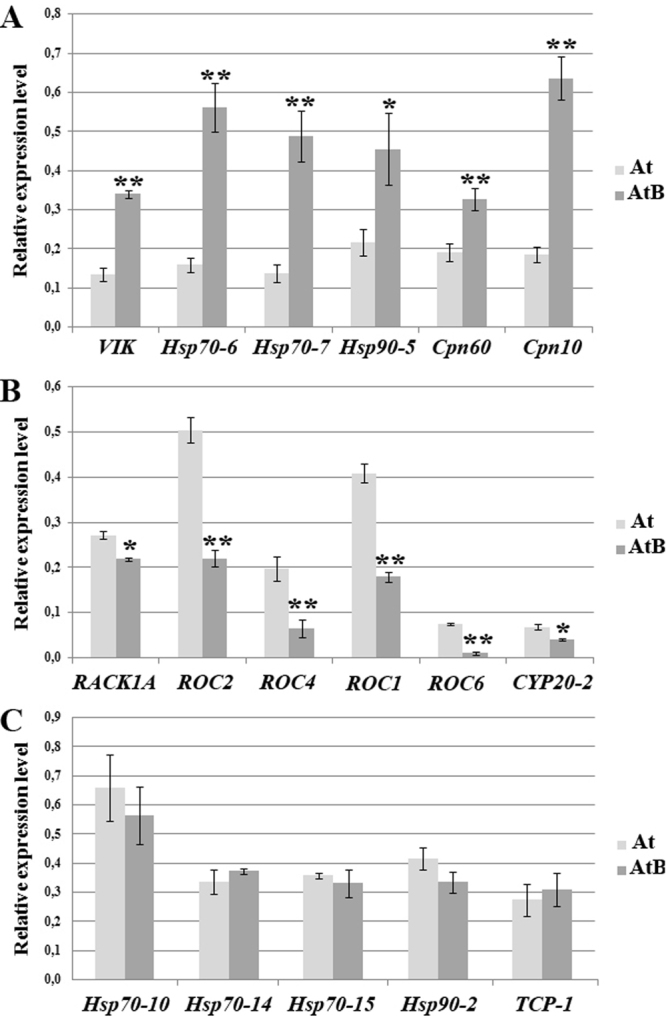
Expression of chaperone genes, *VIK* and *RACK1A* in *Arabidopsis* normal and *rolB*-transformed calli. RNA samples were isolated from callus cultures during the linear phase of growth (20–22 days). qPCR data (mean ± standard error) were summarized from two biological and three technical replicates. Asterisks indicate statistically significant differences of means (*P < 0.05; **P < 0.01), Fisher’s LSD.

To confirm the results of the proteomic analysis, qPCR was performed to detect expression of genes corresponding to six up-regulated proteins, six down-regulated proteins and five proteins which abundance was not changed in *rolB*-expressing *Arabidopsis* calli (Fig. 1). The activation of *VIK*, *Hsp70-6*, *Hsp70-7*, *Hsp90-5*, *Cpn60* and *Cpn10* genes in AtB callus culture was consistent with the proteomic data (Table 1, Fig. 1A). Expression of *RACK1A*, *ROC2*, *ROC4*, *ROC1*, *ROC6* and *CYP20-2* was decreased in *rolB*-expressing cells (Fig. 1B). In agreement with the proteomic data, no significant differences were observed in expression levels of the *Hsp70-10*, *Hsp70-14*, *Hsp70-15*, *Hsp90-2* and *TCP-1* genes in At and AtB calli (Fig. 1C). Thus, the gene-expression data were in accordance with proteomics data.

### Network Reconstruction and Analysis

**Figure 2:**
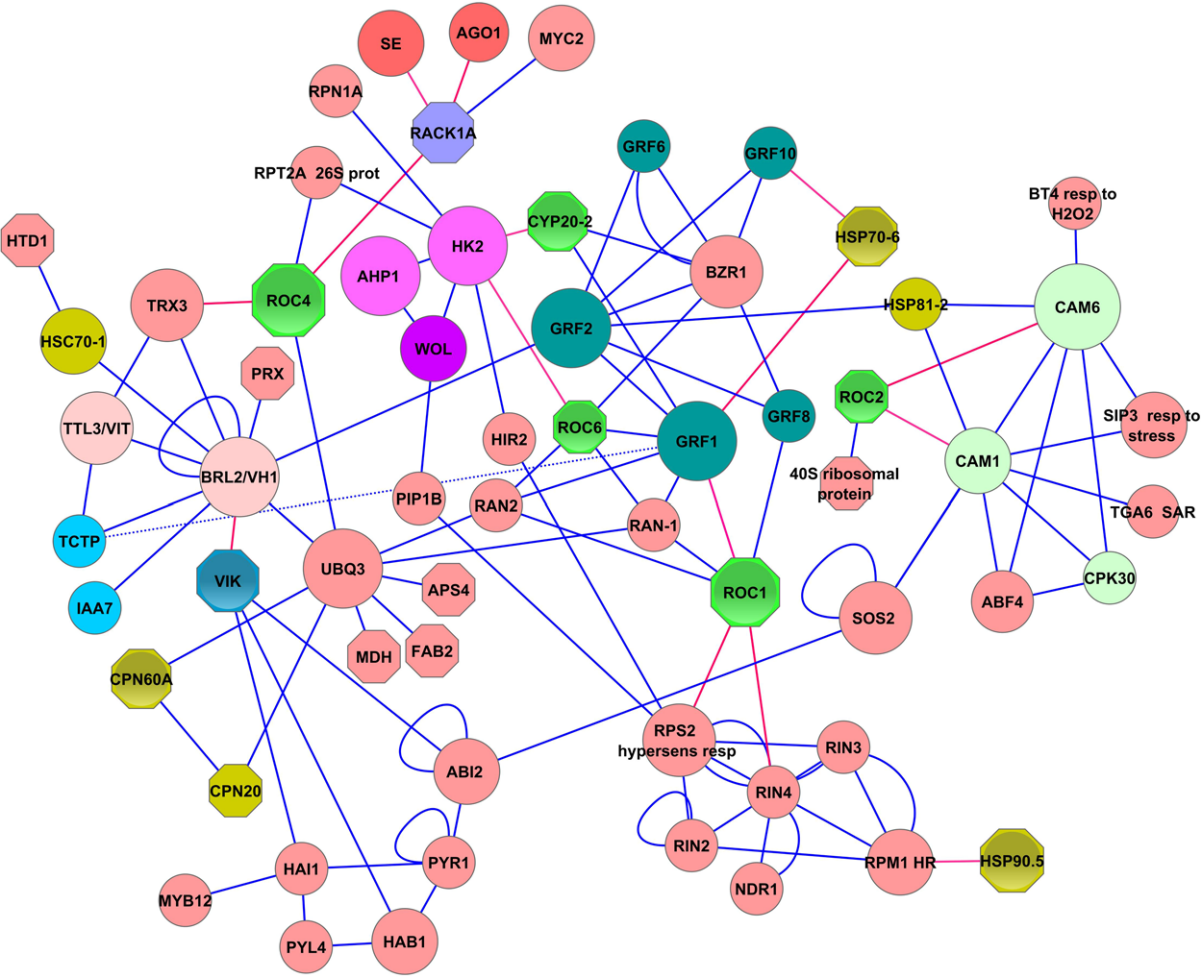
General presentation of changes in the *Arabidopsis* protein signaling network caused by expression of the *rolB* gene. The octahedrons represent proteins whose expression was changed by *rolB*. Heat shock proteins are shown in brown-green and cyclophilins in bright green. The most important interactions are indicated by red lines. The basic signaling modules are as follows (top to bottom): SE and AGO1 (red nodes) represent members of the miRNA processing machinery (the complete subnetwork is presented in^32^). The violet circles HK2, AHP1 and WOL represent core components of the cytokinin signaling network. To their right, a large cluster of general regulatory factors (GRFs) is highlighted in green. In the central part of the figure on the left, the hub proteins BRL2/VH1 and TTL3/VIT (brassinosteroid and auxin signaling) and CAMs on the right (calcium signaling) are presented. The RPM1-RPS2 signaling module is located at the bottom right of the figure. The interactions of VIK with the protein phosphatases HAI1, HAB1 and ABI2 indicate possible links of VIK with abscisic acid signaling and chromatin-remodeling complexes^82^. BZR1, brassinazole-resistant 1; GRFs, growth-regulating factors; HSPs, heat shock preteins; RANs, RAN GTPase-activating proteins; ROCs, rotamase cyclophilins; RACK1A, receptor for activated C kinase 1A; RPT2A, 26S proteasome AAA-ATPase subunit; RPN1A, 26S proteasome regulatory subunit S2 1 A; SE, Serrate; AGO1, Argonaute 1; MYC2, transcription factor MYC2; CYP20-2, CYCLOPHILIN 20-2; HK2, histidine kinase 2; AHP1, histidine-containing phosphotransmitter 1; WOL, histidine kinase 4; PIP1B, aquaporin PIP1-2; TCTP, translationally controlled tumor protein; BRL2/VH1, serine/threonine-protein kinase BRI1-like 2; PRX, PEROXIDASE; TRX3, thioredoxin H3; TTL3/VIT, tetratricopetide-repeat thioredoxin-like 3/VHI-interacting TPR containing protein; HSC70-1, heat shock cognate protein 70-1; HTD1, heat stress tolerant DWD 1; UBQ3, polyubiquitin 3; CPNs, chaperonins; MDH, malate dehydrogenase; FAB2, fatty acid biosynthesis 2; APS4, sulfate adenylyltransferase; VIK, VH1-interacting tetratricopeptide repeat (TPR)-containing protein; HAI1, highly ABA-induced PP2C; MYB12, transcription factor MYB12; PYL4, abscisic acid receptor PYL4; HAB1, protein phosphatase 2 C 16; ABI2, protein phosphatase 2C 77; PYR1, abscisic acid receptor PYR1; SWI3B, chromatin remodeling complex subunit B; HIR2, hypersensitive-induced response protein 2; RPS2, disease resistance protein RPS2; RINs, E3 ubiquitin protein ligases; RIN4, RPM1 interacting protein 4; NDR1, non-race specific disease resistance protein 1; RPM1, disease resistance protein RPM1; SOS2, CBL-interacting serine/threonine-protein kinase 24; CAMs, calmodulins; BZIPs, basic leucine-zippers; BZO2H1, basic leucine zipper 10; ABF4, ABA-responsive element binding protein 4; CPK30, calcium-dependent protein kinase 30; BT4, BTB and TAZ domain protein 4; SIP3, CBL-interacting serine/threonine-protein kinase 6; TGA6, transcription factor TGA6; NPR1, nonexpresser of PR genes 1.

To create a subnetwork of signaling components affected by RolB, we used our previous reconstruction of the *Arabidopsis* interactome^32^, as well as algorithms for construction of subgraphs and validation of small subnetworks^35,36^. Our analysis indicated high level of integrity of the subnetwork presented in Fig. 2. Deleting the individual nodes indicated by octagons (affected by RolB) eliminated the subnetwork. Removing nodes that are not directly related to octagons does not destroy the network (in this case, the network turns out to be simpler). As can be seen from Fig. 2, the network shows the perturbations of the proteome but does not show input nodes. Although it is impossible at present to determine the primary targets of the oncoprotein, the reconstruction is useful for creating a working model.

#### Cyclophilins

ROC1 (CYP18-3): As a first step in the reconstruction of signaling components affected by RolB, we began to reconcile ROC1 interactions (Fig. 2). The abundance of ROC1 in *rolB*-transformed cells is significantly decreased (Table 2). Via RIN4, ROC1 is connected to the RPM1-RPS2 signaling module^46^ that controls effector-triggered immunity. Another consequence of ROC1 deficiency might be perturbations in the expression of ROC1-associated proteins such as RAN1 and RAN2 (RAN GTPase-activating proteins, Fig. 2) as well as the 14-3-3 proteins GRF1 and GRF8 (general regulatory factors). RANs mediate protein import into nuclei and the cellular response to salt stress. The interaction of ROC1 with GRFs was demonstrated previously^47^, but neither the exact mechanism of this interaction nor its outcome is known.

ROC2 (CYP19-3): ROC2 physically interacts with calmodulins (CAMs) and thus affects a broad array of reactions controlled by CAMs^48^. These include the response to stress (mediated by the CBL-interacting serine/threonine-protein kinase 6 (SIP3), BTB and TAZ domain protein 4 (BT4) and basic leucine-zipper proteins; Fig. 2) and induced systemic resistance (mediated by TGA6 and NPR1). It is likely that in this manner, i.e., via ROC2-CAM interactions, *rolB* also exerts its modulating effect on calcium-dependent protein kinases^49^.

ROC4 (CYP20-3): ROC4 connects redox and light signals to cysteine biosynthesis and stress responses in chloroplasts^50^ and is known to be a key effector protein that links hormone signaling to amino acid biosynthesis and redox homeostasis during stress responses^51^. The important interactions of ROC4 include its interaction with the 26S proteasome subunit RPT2A and with RACK1A (Fig. 2). RPT2A controls the meristematic activity in roots and shoots^52^. Both RPT2A and RACK1A mediate crosstalk between developmental and defense signaling pathways in plants^44,45,53,54^. Reduction of the concentration of ROC4 in transformed cells should ultimately lead to a change in the immune status of the cells. Unfortunately, the precise mechanism of the ROC4-RPT2A interaction or ROC4-RACK1A interaction is unknown.

RACK1 is a WD-40-type scaffold protein that is conserved in eukaryotes and plays regulatory roles in diverse signal transduction and stress response pathways^44^. RACK1A ensures the accumulation and processing of some pri-miRNAs, directly interacting with SERRATE and the AGO1 complex^55^. These interactions explain recent data indicating the active involvement of *rolB* in the modulation of expression of core components of the miRNA processing machinery, including SERRATE and AGO1^56^.

ROC4 also interacts with TRX3 (thioredoxin)^57^. TRX3 controls the abundance of numerous proteins that are involved in a wide variety of processes including the Calvin cycle, metabolism, photosynthesis, defense against oxidative stress and amino acid synthesis^57^. Again, the precise function of the ROC4-TRX3 interaction is unknown (see also the interaction data presented at http://www.ebi.ac.uk/intact/interaction/EBI-449668;jsessionid=BC14D657B31C0FA84F73F7E9DC43F683). However, because TRX3 has a dual function as a disulfide reductase and a molecular chaperone^58^, decreased ROC4 abundance could diminish ROC4-TRX3 interactions and thus TRX activity. Indeed, many of the proteins whose expression is increased in *rolB*-expressing cells (Table 1) are under the control of TRX3^59^. These include ascorbate peroxidases, glutathione S-transferase DHAR, glutathione S-transferase F6, alanine aminotransferase and others.

ROC6 (CYP19-2) and CYP20-2: The cyclophilins ROC6 (CYP19-2) and CYP20-2 interact with the transcriptional repressor BZR1 and the cytokinin signaling system (Fig. 2). The interaction between CYP20-2 and BZR1 is presently considered important in the regulation of flowering^60^. BZR1 modulates ovule initiation and development by monitoring the expression of genes related to ovule development. The HK2 (histidine kinase 2) cytokinin receptor, together with the histidine-containing phosphotransferase protein AHP1 and the histidine kinase WOL, regulates many developmental processes including meristematic activity, cell division, chlorophyll content, root growth and shoot promotion (TAIR annotation). Reprogrammed reproductive fate of the ovule, decreased chlorophyll content, lateral root growth and shoot promotion are characteristic traits of *rolB*-transformed plants^7,8,61^. Therefore, the CYP19-2:CYP20-2–HK2/BZR1 interactions provide evidence in favor of the involvement of *rolB* in cytokinin signaling and may explain the numerous cytokinin-dependent morphological alterations observed in *A. thaliana rolB*-transformed plants^61^.

#### VH1-interacting kinase (VIK)

Expression of *rolB* in *Arabidopsis* calli led to the activation of several regulatory proteins. We found increased expression of the VH1-interacting kinase (VIK) in *rolB*-transformed cells (Table 1). VIK participates in the regulation of the hub protein VH1/BRL2, facilitating the diversification and amplification of signals perceived by VH1/BRL2^62^. VH1/BRL2, in turn, interacts with TCTP (translationally controlled tumor protein), a general regulator required for the development of the entire plant, and with IAA7 (auxin-responsive protein IAA7), one of the members of the AUX/IAA family of auxin-induced transcriptional regulators. VIK is involved in the auxin-activated signaling pathway, the defense response to fungi, the negative regulation of programmed cell death, regulation of the plant-type hypersensitive response and responses to cold and water deprivation (TAIR annotation). Many of these responses have previously been shown to occur in *rolB*-expressing cells. RolB perturbs the auxin signaling pathway^1^, activates the defense response to fungi^12^, negatively regulates programmed cell death^10^, ensures higher resistance to salinity, cold and water deprivation^11^ and causes symptoms that closely resemble systemic acquired acclimation^9^. However, we could not find a relationship between VIK and cyclophilins either in our reconstructions or the literature.

#### Auxins and cytokinin signaling

It is generally accepted that *rolB*-induced modification of hormone signaling causes developmental abnormalities in transformed plants. The interaction of *rolB* with the protein module VIK-VH1/BRL2-(TCTP; IAA7) (Fig. 2) offers a plausible explanation of the mechanism by which *rolB* modulates auxin signaling. TCTP is a central mediator of auxin homeostasis and root development^63^; modification of its activity might be essential for the manifestation of many *rolB*-induced traits. Moreover, the function of TCTP in regulating cell division is part of a conserved growth regulatory pathway that is shared by plants and animals^64^, further confirming the idea that plant oncogenes affect ancient regulatory mechanisms. TCTP interacts with GRF1; modulation of its activity by *rolB* might also occur more directly via ROC1-GRF1-TCTP interaction (Fig. 2). IAA7 is connected with the expanded auxin subnetwork (27 proteins; the complete auxin network is presented in ref.^32^). IAA7 mediates not only the response to auxin but also gravitropism. Lessening of gravitropism is a well-known effect of *rolB*^4^.

It is clear that modification of auxin signaling in *rolB*-expressing cells is closely connected to the modification of cytokinin signaling. One pathway by which *rolB* might affect cytokinin signaling involves the interaction of ROC6 and CYP20-2 with HK2. This interaction affects the central cytokinin signaling module HK2-AHP1-WOL (indicated by the violet circles in Fig. 2). Thus, promising interactions for further investigation of the modification of auxin/cytokinin pathways in *rolB*-transformed cells include VIK-VH1/BRL2-(TCTP; IAA7), ROC1-GRF1-TCTP and ROC6;CYP20-2–HK2. It is very likely that auxin signaling is affected by cyclophilins in *rolB*-transformed cells; recent studies have shown a pivotal role of cyclophilins in auxin signaling and lateral root formation that includes perturbation of the activity of auxin-responsive Aux/IAA family proteins^65,66^. Certainly, these predictions must be further confirmed by experimental evidence.

## Discussion

### Primary Metabolism

Some enzymes of primary metabolism were highly activated in *rolB*-transformed cells, whereas some decreased in abundance. At first, these observations seem contradictory. However, we found that most of the enzymes that were hyper-activated in *rolB*-transformed cells have been shown to be highly responsive to various types of stress^59^ (Table 1). In general, *rolB* suppresses primary metabolism and activates anti-stress defense pathways in cells.

### Chaperonin Family Proteins

Hsp70s are highly conserved in eukaryotes, and some their functions are conserved in animals and plants. In animals, overexpression of Hsp70 was found to confer tumorigenicity and provide a selective survival advantage to tumor cells due to its ability to inhibit multiple pathways of cell death, including apoptosis^67^. In the case of the *rolB* gene, we can see a similar picture, i.e., increased abundance of some Hsp70 proteins (Table 1) and inhibition of programmed cell death^10^. Therefore, *rolB* may function to provide favorable conditions for tumor growth after T-DNA integration. Only chloroplastic forms of Hsp proteins such as Hsp70-6, Hsp70-7, Hsp90-5/CR88 (synonym: Hsp88.1), 20-kDa chaperonin and chaperonin 60 subunit α1 were upregulated in *rolB*-transformed cells. Expression of genes encoding these proteins was also upregulated (Fig. 1). Indeed, recent data have shown the higher expression of genes encoding chloroplast heat-shock proteins in *rolB*-transformed tomato plants, compared with normal plants^68^.

It is presently unclear which reactions represent the direct action of RolB and which reactions compensate for this action. Presently, we assume that increased expression of chloroplastic heat shock proteins (Hsp70-6 and Hsp70-7, Hsp90-5, 20-kDa chaperonin and chaperonin 60 subunit α1) in *rolB*-transformed calli represents some kind of compensatory reaction. We propose the following development of events after the transformation. Basal levels of chaperones facilitate normal protein folding and guard the proteome against misfolding and aggregation. Increased expression of chaperones in normal *Arabidopsis* cells subjected to stress, which has been reported many times previously, is an adaptive response that enhances cell survival. The increased expression of chaperone proteins in *rolB*-transformed cells reflects the efforts of these cells to maintain homeostasis. These chaperone proteins also help tumor cells balance changes in cell biochemistry.

The enhanced expression of chaperonin family proteins in *rolB*-transformed calli can be linked with the decreased expression of cyclophilins CYP18-3 (ROC1), CYP19-2 (ROC6), CYP19-3 (ROC2), CYP20-2 and CYP20-3 (ROC4). Little is known about the functional connection of heat shock proteins with cyclophilins in plants^69^, but in animal and human studies, connections of this type have been demonstrated^20^. These interactions are critical in establishing tumor phenotypes through the disturbance of processes involved in protein folding, trafficking and degradation. Whereas these investigations are of high importance for human biology^20^, they are presently almost unknown for plant biology and represent an emerging (and intriguing) topic for understanding the formation of tumor phenotypes in plants.

Plant cells transformed with the *rolB* gene tolerate high temperatures^11^. Many properties of *rolB*-transformed cells resemble those of heat-acclimated plants, including inhibition of plant cell death, Hsp activation and induction of ascorbate peroxidases and other defense enzymes^70^. However, a fundamental difference is that the expression of cyclophilins is increased in heat-acclimated plants^70^ but decreased in *rolB*-expressing cells. Taken together, our results indicate that *rolB* affects the expression of chaperone-type proteins such as heat-shock proteins and cyclophilins. These chaperones seem to regulate several layers of developmental and defense processes and potentially can affect many components of the *Arabidopsis* signaling system, including the RPM1-RPS2 signaling module, auxin and cytokinin signaling, the calcium signaling system and secondary metabolism.

### Effector-Triggered Immunity

According to the zig-zag model of the plant immune system^71^, pathogens have evolved virulence factors that promote pathogen growth by suppressing pattern-triggered immunity (PTI). To counteract the action of specific pathogen effectors, plants have evolved effector-triggered immunity (ETI)^72^. In Arabidopsis, the ETI receptor RPM1 is activated by phosphorylation of the RPM1-interacting protein RIN4. During activation of the RPS2 pathway, RPS2 physically interacts with RIN4^73^. RPS2 initiates signaling based upon perception of RIN4 disappearance and induces plant resistance^73^.

The most probable scenario for *rolB* action is its primary effect which is inhibition of ROS, apoptosis and eventually cell immunity. However, *rolB*-transformed cells counteract this action in various ways. The first way is ROC1 suppression. Because ROC1 suppresses RPM1/RIN4 immunity in a PPIase-dependent manner^46^, it can be assumed that *rolB*-transformed cells, by suppressing ROC1, attempt to maintain a constitutively activated process that resembles ETI. Therefore, the final effects of *rolB* gene expression resemble ETI more than PTI. It is likely that RolB partially mimics the action of nucleotide-binding/leucine-rich-repeat (NLR) receptors that are necessary for ETI^72,74^.

On the other hand, RPM1 is an Hsp90-5 client protein^75^ (Fig. 2). Hsp90-5, together with cofactors, ensures dynamic interactions in the module Hsp90-5-PBS2/RAR1-SGT1, which regulates the stability and function of RPM1^75^. Therefore, the current hypothesis is that *rolB* controls the RPM1-RPS2 signaling module in two ways: via ROC1-RIN4 and via Hsp90-5–RPM1 interactions.

### RolB, Cyclophilins and RACK1A

Ito and Machida recently suggested that plant T-DNA oncogenes change the epigenetic status of the host chromatin through intrinsic histone chaperone activity^17^. Indeed, in both plants and animals, cyclophilins acting as PPIases and chaperones alter transcription by altering chromatin structure and by other mechanisms that include the recruiting of chromatin- and histone-modifying enzymes^76^. Another possible effect of cyclophilin silencing in *rolB*-expressing cells is silencing of RACK1A, an important protein that regulates the small RNA (miRNA and short interfering RNA)-processing machinery. Therefore, the action of the *rolB* gene could be similar to that of the *6b* gene, the product of which targets key components of the small RNA processing machinery, namely both the DCL1-SE-HYL1 and RISC/AGO1 complexes^77^. Intriguingly, RACK1 suppression promotes gastric cancer by modulating the expression of miRNAs^78^. RACK1 inhibition may be important for *rolB*-mediated tumor progression in plants.

### Secondary Metabolism

The mysterious ability of *rolB* to greatly activate secondary metabolism in transformed cells has been known for many years^13^. It was recently shown that expression of *rolB* in *Arabidopsis thaliana* calli leads to the activation of genes encoding secondary metabolism-specific MYB and bHLH transcription factors^15^. Accordingly, a higher transcript abundance of corresponding biosynthetic genes related to these factors was detected. The effect of *rolB* on the expression of transcription factors was highly specific; for example, *rolB* did not induce *MYB111* or *PAP1* expression and caused the conversion of *MYB* expression from cotyledon-specific to root-specific patterns^15^.

It should be noted that none of the regulatory proteins described in the present work whose expression was changed by *rolB* gene activity can be attributed to the common secondary metabolism activator pathways described earlier for *Arabidopsis*^32^. The *rolB* gene most likely does not affect secondary metabolism directly; its effect is more likely a part of general defense reactions. We suggested three signaling modules by which *rolB* might influence secondary metabolism: ROC4-RACK1A → MYC2 (MYB2-TT8; JAZ1-TT8); (VIK-HAI1-HAB1-ABI2)-MYB12 and ROC2-(CAM-CDPK) (Fig. 2). The first of these is based on the MYB2 signaling module, which connects secondary metabolism with hormone (JA, auxin, cytokinin and ethylene) signaling^32^. The second represents the connection between secondary metabolism and abscisic acid, which is mediated by HAI1-MYB12 interactions^79^. The third, ROC2-(CAM-CDPK) module, represents a pathway of secondary metabolism activation known as activation through calcium-dependent protein kinases^80^. Considering the observation that *rolB* is a more powerful activator of secondary metabolism than a constitutively expressed *CDPK* gene^81^, we suggest that more than one mechanism is involved in its activator function.

## Electronic supplementary material

Dataset 1

Dataset 2

Dataset 3

Supplementary information
