# Supplementary material for: The rolB plant oncogene affects multiple signaling protein modules related to hormone signaling and plant defense
Source: Sci Rep. 2018 Feb 2;8:2285. doi: 10.1038/s41598-018-20694-6 (PMC5797197; doi:10.1038/s41598-018-20694-6)

SUPPLEMENTARY INFORMATION

The *rolB* plant oncogene affects multiple signaling protein modules related to hormone signaling and plant defense

Victor P. Bulgakov[1,2*], Yulia V. Vereshchagina[1], Dmitry V. Bulgakov[1] , Galina N. Veremeichik[1], Yuri N. Shkryl [1]

[1] *Institute of Biology and Soil Science, Far Eastern Branch of the Russian Academy of Sciences, 159 Stoletija Str., Vladivostok, 690022, Russia*

[2] *Far Eastern Federal University, Vladivostok, 690950, Russia*

* bulgakov@ibss.dvo.ru

Contents

Supplementary Table 1: List of primer sequences used for qPCR analysis …………………………..... 2

Supplementary Table 2: Comparison of the strength of *rolB* expression in *rolB*-transformed *A. thaliana* callus lines transformed with the *rolB* gene expressed under the control of the 35S CaMV promoter ....……………………………………………………………………..... 4

Supplementary Figure S1: Typical 2-DE maps used in the present investigation ……………………. 4

Supplementary Figure S2: Comparison of the protein expression level on 2D gels ………………..... 6

Fig. S2-1: Heat shock 70 kDa protein 6, heat shock 70 kDa protein 7, chaperonin 60 subunit alpha 1, heat stress tolerant DWD 1 …………………………………………………..……………… 6

Fig. S2-2: Peptidyl-prolyl cis-trans isomerase ROC1 (CYP18-3, CP18C_ARATH) ……….…. 6

Fig. S2-3: Receptor for activated C kinase 1A, RACK1A (GBLPA_ARATH) …………………7

Supplementary Table 1: List of primer sequences used for qPCR analysis.

№	UniProtKB code	Genbank accession number	Direct primers, (5′-3′)	Reverse primers (5′-3′),
1	HSP7J_ARATH	AY079015	TCCCAAGTATGCCACACCG	GCTGGTTGCTCTGGCTCACTC
2	HSP7O_ARATH	AY079015	CTCATCCGCCTGCTCACCTC	CTTCCCAAGTATGCCACACC
3	HSP7P_ARATH	AY065177	GAGGCAGCTGTGTCTAATG	CTCTTGCCCTCATCCGCCTG
4	HS902_ARATH	AY128805	GAAGAAGAAAGAAGAGCTCAAG	GAAACCAGAAGTGAGAAGAG
5	TCPD_ARATH	BT003418	CTATCGCCATTGTGACTGAACTC	GAAACTACCACACTACCTCACAG
6	GBLPA_ARATH	AY035007	CTCTTGGTGGCAGCAGGAC	GTTGTTTTGCTGTGGGATTTG
7	CP19C_ARATH	AY072128	CTTTCACCGTATAATCCCAG	CCTGTCAAGATCAACCCACCC
8	CP20C_ARATH	AF325026	CAATGCATCGGTCAATAGTG	CTGGTGAGAAGAAATACGGG
9	CP18C_ARATH	AY093227	GGAACGGAACAGGCGGTGAG	AAGAGACACCACAATGACGAC
10	CP19B_ARATH	BT025527	CACCGACAAGACACCAAAGACG	CCACCTGACCAAACACCAC
11	PNSL5_ARATH	AY568517	CATTCCATCGTGTCATCAGAG	CACTTCCATTCCTTCTATTAC
12	Q9XI87_ARATH	BT026491	GTTGGAGACTTTGGACTTAG	GATACATGTTTGGCTGCTTC
13	HSP7F_ARATH	AY072138	CAACCCAAGAAATCAAAGAC	GTTTTCTCATTGGCTGTCTG
14	HSP7G_ARATH	BT000919	CAACTCAAGAAATCAAAGACAC	CGTCAATCACATCACCGCCG
15	HS905_ARATH	AF436826	CTTCTCCTTGTGTGCTTGTC	CTTGTTGCTTCAGTGCTCTC
16	CPNA1_ARATH	BT002441	CATTTGCTGCTATCGAGGAAG	CGCACATCTTGTCACTTTAGC

17	CH10C_ARATH	AJ010818	CCGAGACTACCAAAGAGAAG	CTAAGAAAGTATAGCCATCAC
18	ACT2_ARATH	NM_001338359	ACTTTCATCAGCCGTTTTGA	ACGATTGGTTGAATATCATCAG
19	UBQ10_ARATH	NM_178968	GGCCTTGTATAATCCCTGATGAAT	AAAGAGATAACAGGAACGGAAACATA

Supplementary Table 2: Comparison of the strength of *rolB* expression in *rolB*-transformed *A. thaliana* callus lines transformed with the *rolB* gene expressed under the control of the 35S CaMV promoter

A. thaliana callus lines	
At (control callus culture)	-
AtB1(*rolB*-transformed callus culture)	0.221 ± 0.006[A]
AtB2(*rolB*-transformed callus culture)	0.561 ± 0.041[B] selected for analysis

Quantitative real-time PCR (qPCR) analysis. RNA samples were isolated from callus cultures (20–22 d) of *A. thaliana*. The data are presented as mean ± standard error from three biological replicates with two technical replicates for each experiment. Different letters (superscript) indicate statistically significant differences of means ($p<0.05$), Fisher's LSD. Dashes indicate the absence of expression.

Supplementary Figure S1: Typical 2-DE maps used in the present investigation

A, C, E: vector-transformed callus cultures of *A. thaliana* and **B**, **D, F:** *rolB*-transformed callus cultures. 500 micrograms of protein was applied to 18 cm long IPG strips (nonlinear pH gradient, pH 3-10), 12% SDS-PAGE gels were used in the second dimension. Coomassie brilliant blue G-250 staining was used for protein detection. Red squares represent the areas shown in greater detail in Supplementary Figure S2.

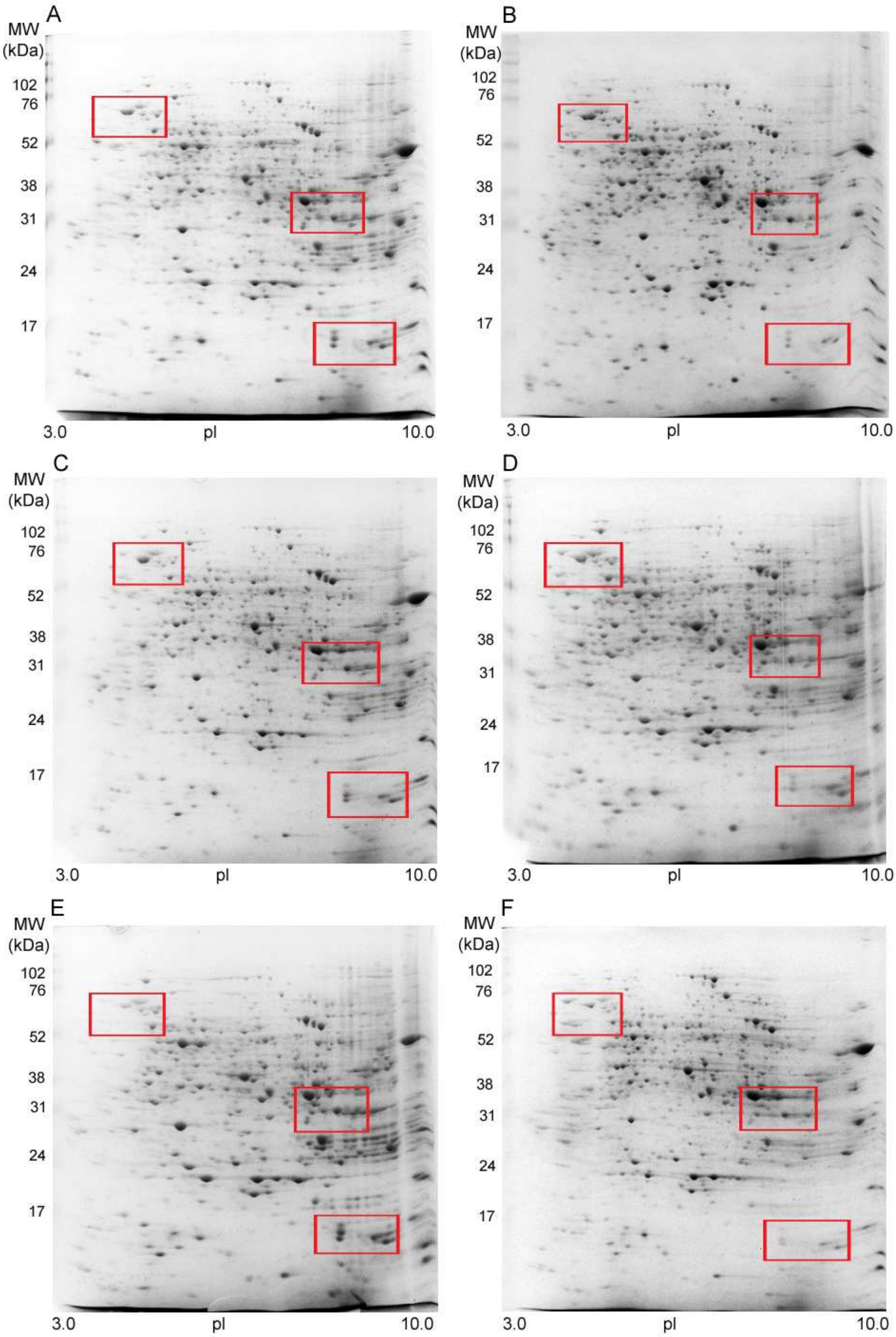
A
B
C
D
E
F
MW (kDa)
102
76
52
38
31
24
17
3.0
pI
10.0

Supplementary Figure S2: Comparison of the protein expression level on 2D gels

Gels were scanned using the PharosFX Plus System. PDQuest 8.0.1 Advanced software (Bio-Rad) was used for analysis of protein spots. Three control and three experimental gels were used in the analysis.

Fig. S2-1: Heat shock 70 kDa protein 6, heat shock 70 kDa protein 7, chaperonin 60 subunit alpha 1, heat stress tolerant DWD 1.

A, control calli; **B,** *rolB*-transformed calli of *A. thaliana*.

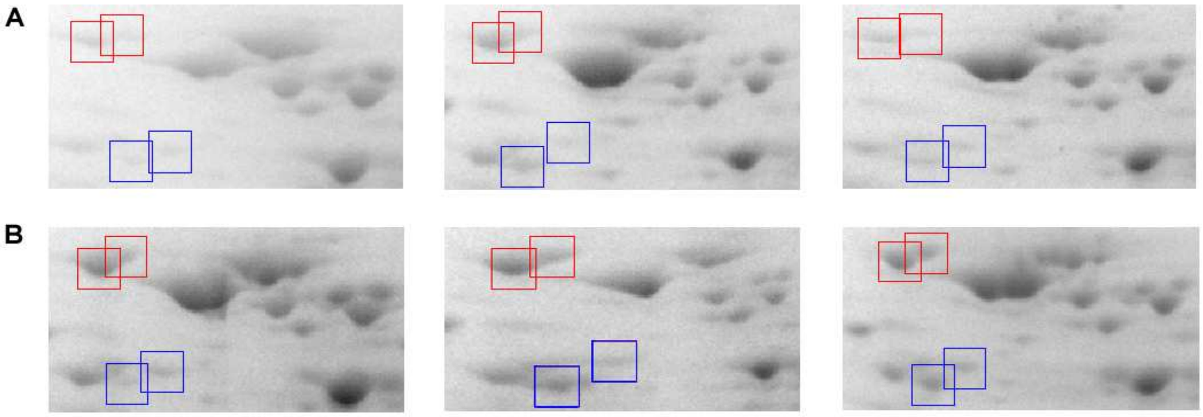

Red square on the left: heat shock 70 kDa protein 6, chloroplastic (HSP7F_ARATH).

Red square on the right: heat shock 70 kDa protein 7, chloroplastic (HSP7G_ARATH).

Blue square on the left: chaperonin 60 subunit alpha 1, chloroplastic, Cpn60 (CPNA1_ARATH).

Blue square on the right: heat stress tolerant DWD 1. Synonym: HTD1/WD-40 repeat family protein (Q9ZUN8_ARATH).

Fig. S2-2: Peptidyl-prolyl cis-trans isomerase ROC1 (CYP18-3, CP18C_ARATH).

A, control calli; **B,** *rolB*-transformed calli of *A. thaliana*

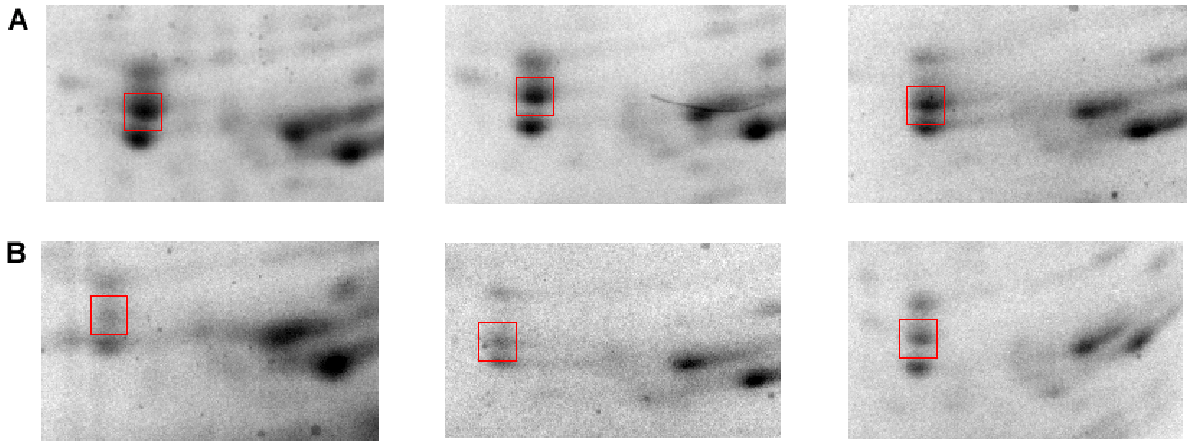

Fig. S2-3: Receptor for activated C kinase 1A, RACK1A (GBLPA_ARATH).

A, control calli; **B,** *rolB*-transformed calli of *A. thaliana*

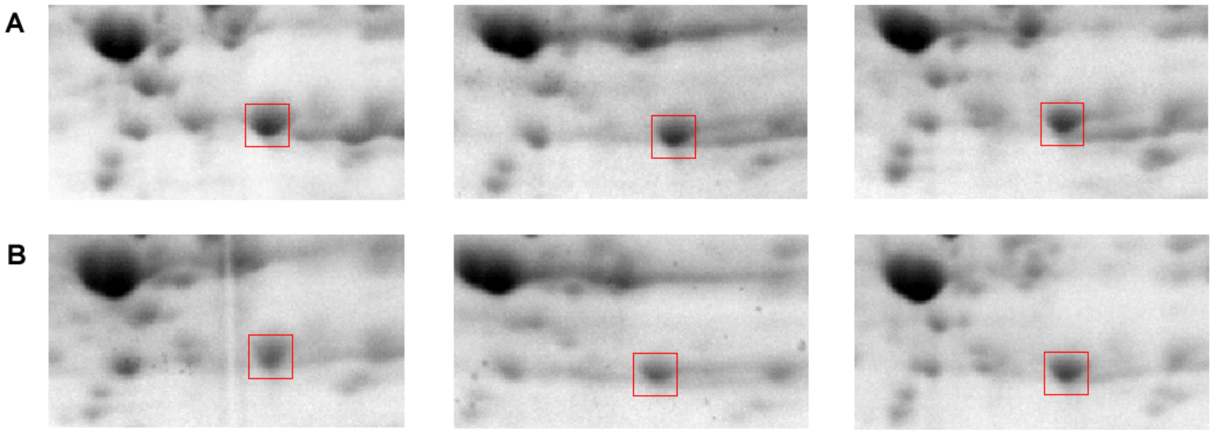

Supplement: Supplementary file 4 — Supplementary information [file 41598_2018_20694_MOESM4_ESM.pdf]
